# Grub polypeptide extracts protect against oxidative stress through the NRF2-ARE signaling pathway

**DOI:** 10.1080/19768354.2021.2018043

**Published:** 2021-12-27

**Authors:** Jingyang Chen, Yingjian Sun, Shan Huang, Hong Shen, Yongjie Chen

**Affiliations:** a Animal Science and Technology College, Beijing University of Agriculture, Beijing, People’s Republic of China; b Beijing Key Laboratory of Traditional Chinese Veterinary Medicine, Beijing University of Agriculture, Beijing, People’s Republic of China; c Central Laboratory, Beijing Obstetrics and Gynecology Hospital, Beijing Maternal and Child Health Care Hospital, Capital Medical University, Beijing, People’s Republic of China

**Keywords:** Grub polypeptide extracts, muscle satellite cells, NRF2-ARE signaling pathway, antioxidant enzymes, oxidative stress

## Abstract

Grub polypeptide extracts (GPEs) have antioxidant effects; however, their underlying molecular mechanisms are unknown. This study explored the antioxidant molecular mechanism of GPE via the nuclear factor-erythroid 2-related factor 2 (NRF2)-antioxidant response element (ARE) signaling pathway in C2C12 muscle satellite cells exposed to oxidative stress. The effects of GPE/or H2O2 on C2C12 were investigated by the MTT (3- (4,5-dimethylthiazol-2-yl)-2,5-diphenyltetrazolium bromide) viability assay and immunofluorescence and small interfering RNA (siRNA) analyses. The cell viability, cell damage, intracellular reactive oxygen species (ROS) levels, and NRF2 signaling pathways related to proteins were measured. GPE significantly increased the antioxidant capacity of cells, evident by increased cell viability and decreased lactate dehydrogenase leakage, DNA damage, malondialdehyde content, and ROS level. GPE also markedly increased mRNA expression levels and activities of antioxidant enzymes including superoxidase 1 and 2, catalase, and glutathione peroxidase. In addition, GPE increased the gene and protein expression of NRF2 and heme oxygenase 1 by promoting NRF2 translocation from the cytoplasm to the nucleus and activating NRF2-ARE signaling pathways. The antioxidant effects of GPE through these signaling pathways were further confirmed by NRF2-specific siRNA silencing. Thus, GPE enhances antioxidant capacity and alleviates oxidative damage of C2C12 cells via the NRF2-ARE signaling pathway.

## Introduction

Reactive oxygen species (ROS) are unavoidable by-products of aerobic metabolism (Schieber and Chandel 2014). Low levels of ROS are necessary for the body to carry out certain biological reactions, including cell signal transduction, cell proliferation and differentiation, adaptation to stress, and metabolic adaptation (Vargas-Mendoza et al. 2019). Normally, ROS are scavenged effectively by the antioxidant system of cells (Martinez-Cayuela 1995). However, when the balance of ROS levels is disturbed, the suddenly high ROS levels damage the lipids, proteins, and DNA in the cells (Kregel and Zhang 2007; Wang et al. 2019). Similar damage can occur during oxidative stress. Cells under oxidative stress appear to misbehave or malfunction, causing a variety of diseases that include cancer and metabolic syndrome (Pellegrino et al. 2011; Thirupathi et al. 2020). Elimination of ROS consists of enzymatic and non-enzymatic antioxidant mechanisms, which are necessary for the body to maintain a delicate intracellular redox balance and reduce or prevent cellular damage caused by ROS (Kregel and Zhang 2007).

Nuclear factor-erythroid factor 2-related factor 2 (NRF2) is a master regulator that mediates antioxidant systems and restores intracellular redox balance. The pivotal role of NRF2 in resistance to oxidative stress mainly involves binding to the promoter of the antioxidant response element (ARE). The binding facilitates the expression of downstream targets, including superoxide dismutase (SOD), catalase (CAT), and glutathione peroxidase (GSH-Px) (Yu et al. 2019). SOD converts O_2_^−^ to hydrogen peroxide (H_2_O_2_) and molecular oxygen (O_2_). CAT and GSH-Px protect the organism from oxidative damage by breaking down H_2_O_2_ to water and molecular oxygen (Mondal et al. 2019). Heme oxygenase-1 (HO-1), another NRF2-ARE pathway-regulated protein, is involved in the catalysis of heme to carbon monoxide, free ferrous iron and biliverdin (Meng et al. 2018). The activation of NRF2-ARE is thought to contribute to reduce oxidative stress and cell death. The level of NRF2 is tightly regulated by Kelch-like ECH-associated protein 1 (KEAP1), a repressor that is responsible for the rapid degradation of NRF2. Under normal conditions, Keap1 binds with NRF2 and sequesters the inactivated NRF2 in the cytoplasm. KEAP1 is easily oxidized in the presence of ROS and changes its conformational state. This results in the release of NRF2 from KEAP1 and its translocation to the nucleus. There, it promotes the transcription of many phase II detoxification enzymes and antioxidant enzymes (Baird and Dinkova-Kostova 2011; Linker et al. 2011; Wei et al. 2019; Zhang et al. 2019).

*Holotrichia diomphalia* Bates is a grub that is a pest to field crops. However, it has numerous pharmacological uses (Dong et al. 2008). The larvae of grubs are a traditional medicine for the treatment of chronic liver cirrhosis, contusion, edema, furuncle, and apoplexy (Oh et al. 2003). The pharmaceutical effects of grub larvae extracts include anticancer, antifungal, anticoagulant, and antibacterial effects (Lee et al. 1994; Dong et al. 2009; Song et al. 2014; Xu et al. 2016). Previous studies have demonstrated that the extracts of insects have antioxidant effects (Zhu et al. 2013). Hong and colleagues demonstrated that grub larvae extract significantly decreased the pulmonary ROS level in mice with ovalbumin-induced asthma (Hong et al. 2019). The extracts of grub polypeptides could also scavenge free radical in vitro (Li et al. 2019.), which prompt that polypeptide extracts from the grub larvae may have an effect on eliminating intracellular ROS directly. However, there are several reports concerning antioxidant molecular mechanisms of GPE. In the present study, mouse-derived C2C12 myoblast cells were used as the model under H_2_O_2_ treatment condition to investigate the pivotal role of GPE through the NRF2-ARE signaling pathway.

Several reports have reported antioxidant molecular mechanisms of grub polypeptide extracts (GPEs). In the present study, mouse-derived C2C12 myoblast cells were used as the model under H_2_O_2_ treatment conditions to investigate the pivotal role of GPE through the NRF2-ARE signaling pathway.

## Materials and methods

### Materials and reagents

C2C12 cells were preserved by our laboratory. Dried grubs were obtained from Tongrentang Chinese Medicine (China). Papain, low melt agarose (A8350), normal melting point agarose (A8201), goat serum (SL038), DAPI (C0065), RIPA buffer (R0010) was purchased from Solarbio (Beijing, China). Dulbecco’s modified Eagle’s media (DMEM) (Sigma–Aldrich, D0822), Tertiary butylhydroquinone (TBHQ) (Sigma–Aldrich, 112941), MTT was purchased from Sigma–Aldrich (M2003, St. Louis, USA). Fetal bovine serum (FBS) was purchased from Gibco Life Technologies (Australia). TRIzol reagent was obtained from Invitrogen (15596018, Shanghai, China). Antibodies against NRF2 (EP1808Y), HO-1 (EP1391Y) and β-actin (ab8227) were purchased from Abcam (Cambridge, UK). Lamin B antibody was obtained from Boster (PB9611, Wuhan, China). SOD (A001-1-2), CAT (A007-1-1), GSH-Px (A005-1-2) and Malondialdehyde (MDA) (A003-1-2) kits were purchased from Nanjing Jiancheng Biological Product (Nanjing, China).

### Preparation of grub polypeptides extracts

The extraction of grub polypeptide was based on the previous study (Li et al. 2019). Briefly, drying grubs were ground into powder, then the water was mixed with the powder in a ratio of 25:1. 6000 U of papain per liter was added to the mixture for 2 h at 55°C to enzymatic hydrolyze it. The extraction rate was 20–30%. Centrifuging the extraction at 4000 rpm for 20 min and getting the crude extracts. Filtering the crude extracts with ultrafiltration tube (Millipore Corporation, USA) and obtaining the grubs polypeptide extracts. Analysis of the content of polypeptide extracts with biuret assay.

### Cell culture and treatment

C2C12 cells were cultured in DMEM containing 10% FBS and 100 μg/ml penicillin/streptomycin in a humidity 5% CO_2_ atmosphere at 37°C. When the density of C2C12 cells was about 90%, the cells were collected and then were divided into four groups, such as control group, H_2_O_2_ group, GPE group and positive (TBHQ) group. The cells of the control group and H_2_O_2_ group were cultured in complete media (DMEM containing 10% FBS) and the cells of the GPE group and positive group were cultured in complete media supplemented with GPE and TBHQ respectively for 24 h, and then except the control group all cells were stimulated with 400 μm H_2_O_2_ for 8 h.

### MTT assay

MTT assay was performed to evaluate the cell viability of C2C12 cells. The cells were incubated with 100 µl of 0.5 mg/ml MTT dissolved with PBS for 4 h in 37°C incubator, then the MTT solution was discarded. 100 µl DMSO was added to dissolve the formazan and measured absorbance at wavelength of 570 nm. Calculation of the relative cell viability with the formula: the relative cell viability =  (the absorbance of test group/the absorbance of control group) *100%.

### The detection of the intracellular ROS

According to the manufacturer’s instruction (S0033S, Beyotime, Beijing, China), the cells were observed with a fluorescence microscope (Olympus, Tokyo, Japan) at the following set: 485 nm excitation and 535 nm emission.

### Activity of antioxidant enzyme and LDH leakage

The activity of SOD, CAT and GSH-Px and the content of MDA was measured according to the manufacturer’s instructions. The leakage rate of lactate dehydrogenase (LDH) was performed according to the standard protocol (C0016, Beyotime, Beijing, China). Absorbance was measured at a wavelength of 490 nm using a microplate reader. Calculation of the leakage rate of LDH with the formula: the leakage rate of LDH = (absorption value of treated samples – absorption value of reference sample)/ (absorption value of maximum enzyme activity – absorption value of reference sample).

### Comet assay

Collecting the cells and mixing them with 0.7% molten low melt agarose. Spreading one drop of the mixture on a fully frosted microscopic slide preheated with 1% normal melting point agarose. Immersing the microscopic slides into lysis solution for 2 h at 4°C after the agarose solidification. Then placing the slides in alkaline solution to relax and denature the DNA. An electrical field was applied (300 mA, 25 V) for 20 min at 4°C to draw negatively charged DNA toward the anode. Staining the slide with 10 ug/ml DAPI and washing them in PBS for three times. The slides were examined under a fluorescence microscope and the resulting images were analyzed (Olympus, Tokyo, Japan). While DAPI emits a blue fluorescent signal, the color of this signal was altered to green in our images to make the effect more obvious.

### Western blot assay

The cells were lysed with RIPA buffer and nuclear proteins were isolated by a nuclear and cytoplasmic protein extraction kit (Beyotime, Beijing, China) according to the manufacture’s instruction. The lysates of cells were separated on SDS-polyacrylamide electrophoresis gels (4% stacking gel and 12% resolving gel) and transferred onto PVDF membranes (Millipore, Massachusetts, USA) using an electroblotting apparatus. Then the membranes were blocked with 4% non-fat milk for 2 h and incubated with primary antibodies including NRF2, HO-1, β-actin and lamin B at 4°C overnight. They were washed with PBST and incubated with secondary antibody for 2 h at room temperature. The proteins were visualized using an ECL chemiluminescence kit (P0018S, Beyotime, Beijing, China). The densitometry of each immunoblot was performed using Image J software (National Institutes of Health, USA). With β-actin as control, the expression of total protein was normalized.

### RT-qPCR assay

Total RNA was isolated from C2C12 cells with TRIzol reagent (Invitrogen, USA). Complementary DNA (cDNA) was generated by reverse transcription of total RNA using HiFi-MmlV cDNA kit (CW0744, CWBIO, Jiangsu, China) and RT-qPCR for gene expression was performed on a Step One Real-time PCR system (Applied Biosystems, USA) by the TB Green Premix Ex Taq (RR420Q, TaKaRa, Dalian, China). The specific primers were as follows: the forward and the reverse primers for SOD1 were 5’-AAGCGGTGTGCGTGCTGAAG-3’ and 5’ TCCTGACAACACAACTGGTTCACC-3’, respectively. The forward and the reverse primers for SOD2 were 5’-ACGCCACCGAGGAGAAGTACC-3’ and 5’-GCTTGATAGCCTCCAGCAACTCTC-3’, respectively. The forward and the reverse primers for CAT were 5’-AGGTGTTGAACGAGGAGGAGAGG-3’ and 5’-AGCGTTGTACTTGTCCAGAAGAGC-3’, respectively. The forward and the reverse primers for GSH-Px were 5’-GGGACACCGCTTACTTTCTC-3’ and 5’-AATCTCTTCATTCTTGCCATTCTC-3’, respectively; the forward and the reverse primers for NRF2 were 5’-GTAGATGACCATGAGTCGCTTGCC-3’ and 5’-CTTGCTCCATGTCCTGCTCTATGC-3’, respectively. The forward and the reverse primers for HO-1 were 5’-ACCGCCTTCCTGCTCAACATTG-3’ and 5’-CTCTGACGAAGTGACGCCATCTG-3’, respectively; the forward and the reverse primers for GAPDH were 5’-AAGAAGGTGGTGAAGCAGGCATC-3’ and 5’-CGGCATCGAAGGTGGAAGAGTG-3’, respectively. GAPDH was used as internal control. The data was analyzed with the 2-ΔΔCt method.

### Immunofluorescence assay

The cells were fixed with 4% paraformaldehyde for 40 min and washed with permeabilization buffer for 30 min (PBS, 0.1% Triton X-100 and 1% BSA) and blocked by 10% goat serum for 1 h at RT. Cells were then incubated with the anti-NRF2 antibody at 4°C overnight, washed with PBST three times and incubated with FITC-conjugated secondary antibody in the dark for 1 h at 37°C next day. Washed the samples three times and incubated with DAPI for 5 min at RT. Samples were observed with a laser scanning confocal microscopy.

### Cell transfection

NRF2 siRNA and control siRNA were purchased from Gene Pharma (Shanghai GenePharma Co., Ltd). The siRNA was transfected into cells according to the manufacturer’s instruction using the Exfect 2000 Transfection Reagent (T101-01, Vazyme, Nanjing, China). For transfection, the cells were seeded in 6-well culture plates and incubated with control siRNA or NRF2 siRNA at 50 nM in a serum-free OPTI-MEM medium. Replace the medium 4 h after transfection. After incubation, the transfected cells were subjected to the treatment for the follow-up experiment. The antisense and sense was designed as 5’-AAUCAAAUCCAUGUCCUGCTT-3’ and 5’-GCAGGACAUGGAUUUGAUUTT-3’, respectively.

### Statistical analysis

Values were presented as the mean ± SD. Data was analyzed from three independent experiments. One-way analysis of variance (ANOVA) was used to compare in the experiments with multiple time points and concentrations. When the comparison between two groups means significant, * or + was used as *P *≤ 0.05. ** or ++ were used as *P *≤ 0.01.

## Results

### Protective effect of GPE on C2C12 cells under oxidative stress

**Figure 1.**
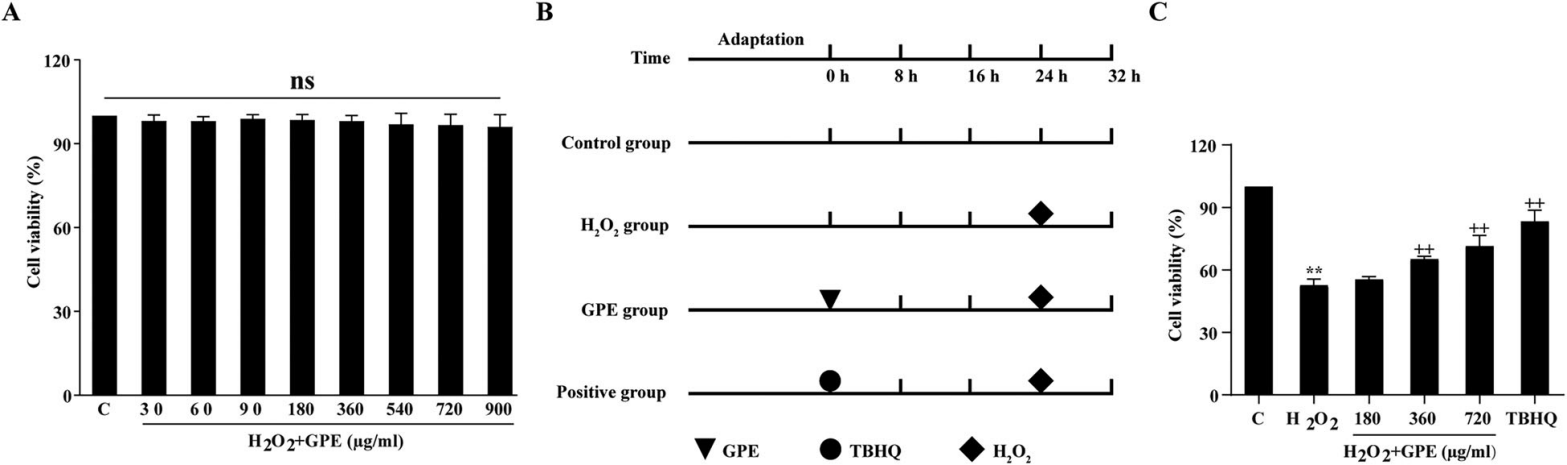
GPE attenuates H_2_O_2_-induced viability inhibition in C2C12 cells. (A) GPE at different concentrations did not affect C2C12 cells viability in the MTT assay. (B) Schematic representation of the different treatments performed. Cells were treated with various concentrations of GPE or 1 μmol/l of TBHQ for 24 h and subsequently stimulated with 400 μM H_2_O_2_ for 8 h or not stimulated. (C) GPE (360 and 720 μg/ml) reduced H_2_O_2_-induced viability inhibition in C2C12 cells, as evidenced by the increased cell viability in the MTT assay. **p < 0.01 compared with the control group, ++*p* < 0.01 compared with the H_2_O_2_-stimulated group.

First, we evaluated the safety of GPE on C2C12 cells. The cells were treated with various concentrations of GPE (30–900 μg/ml) for 24 h. No significant cellular toxicity for different doses of GPE was observed with C2C12 cells in the MTT viability assay (Figure 1(A)).

To examine the effect of GPE on C2C12 cells against oxidative stress, the cells were untreated or treated with GPE for 24 h and then stimulated with 400 μM H_2_O_2_ for another 8 h (Figure 1(B)). The viability of the cells in the H_2_O_2_ group sharply decreased compared with that of cells in the control group. The viability of cells pre-treated with GPE was significantly increased compared with that in the H_2_O_2_ group (Figure 1(C)). These results indicate that the GPE pre-treatment of C2C12 cells could protect the cells from H_2_O_2_ cytotoxicity.

### GPE reduces ROS generation in C2C12 cells

**Figure 2.**
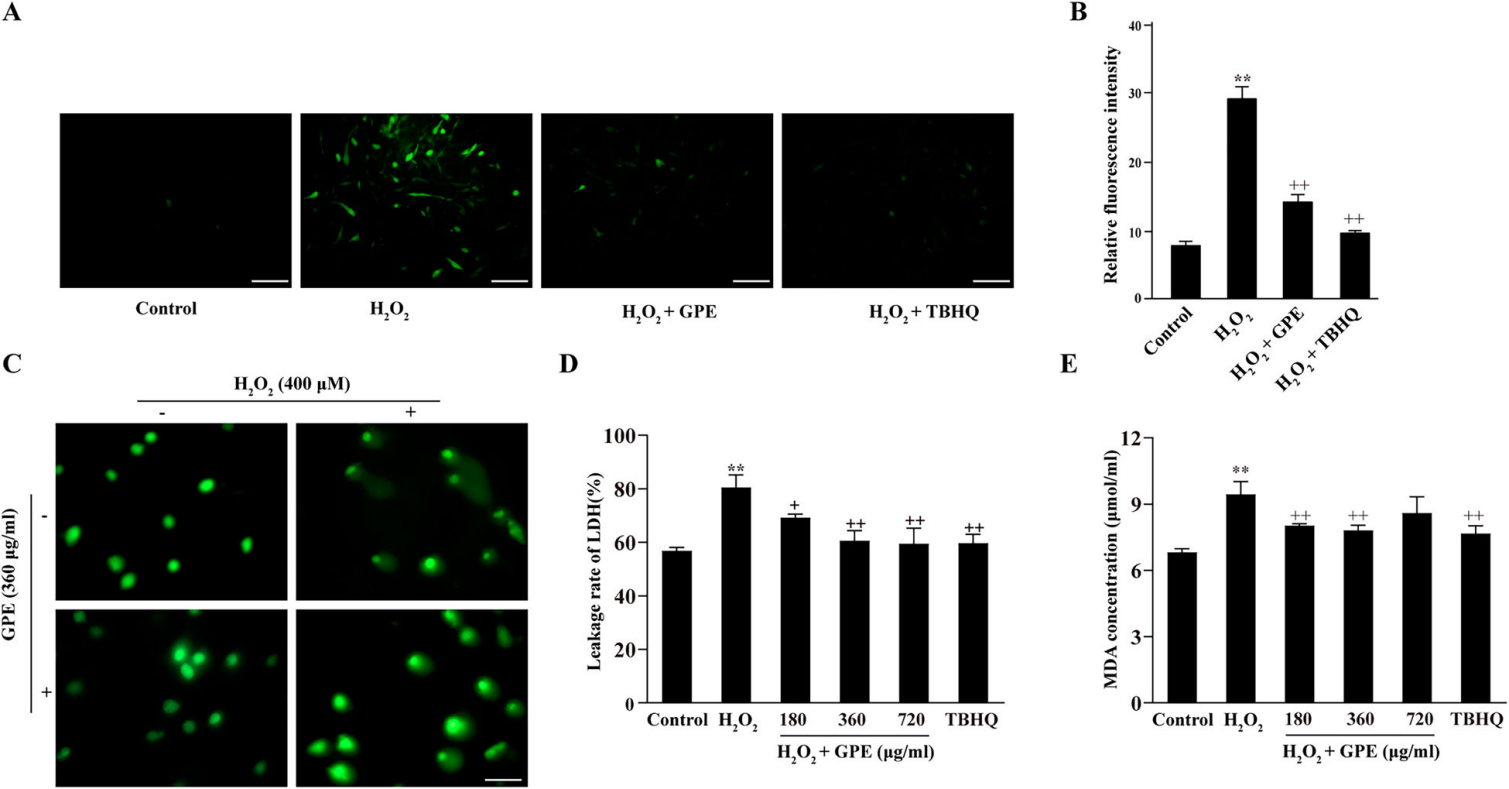
Reduction of H_2_O_2_-mediated DNA damage by GPE in C2C12 cells. (A) ROS generation was observed by the DCFH-DA assay. Scale bar = 100 μm. (B) Relative fluorescence intensity was calculated with the following formula: relative fluorescence intensity = IOD/area. **p < 0.01 compared with the control group, ++*p* < 0.01 compared with the H_2_O_2_-stimulated group. (C) Comet assay was used to analyze cellular DNA damage. Scale bar = 100 μm. (D) LDH concentration measured with an LDH cytotoxicity assay kit. (E) MDA concentration in each group measured with an MDA assay kit. **p < 0.01 compared with the control group, +*p* < 0.05 and ++*p* < 0.01 compared with the H_2_O_2_-stimulated group.

The generation of ROS from C2C12 cells was measured by the Dichloro-dihydro-fluorescein diacetate (DCFH-DA) assay. The intracellular ROS content in cells stimulated with H_2_O_2_ increased significantly compared with that in cells in the control group (Figure 2(A)). However, compared to the ROS level of C2C12 cells exposed to H_2_O_2_, that of cells pre-treated with GPE decreased markedly and was similar to the level in the positive control group (Figure 2(A)). The relative fluorescence intensity measured in the four groups also demonstrated that GPE decreased the intracellular ROS level induced by H_2_O_2_ (Figure 2(B)). These results indicate that GPE has antioxidant effects on C2C12 cells.

### GPE reduces C2C12 cell damage

To evaluate the antioxidant effect of GPE, the comet and spectrophotometry assays were used to analyze the fluorescence intensity in the tails of the comet-like structures, leakage rate of LDH, and the intracellular MDA content. As shown in Figure 2(C), compared with control cells, cells exposed to H_2_O_2_ displayed increased fluorescence intensity in the tails of the comet-like structures, which indicated increased DNA damage in C2C12 cells. An obvious difference was observed between the cells pre-treated with GPE and untreated cells. The dwindled tails displayed by the GPE pre-treated cells confirmed that GPE could remarkably reduce the DNA damage caused by H_2_O_2_ stimulation. Intracellular enzymes like LDH leak from cells when the cell membrane is damaged. The extracellular content of LDH reflects the degree of cell membrane damage. As shown in Figure 2(D), GPE reduced the leakage of LDH from C2C12 cells and lessened cell membrane damage caused by the oxidative stress. MDA is the most commonly used indicator of lipid peroxidation caused by ROS. Analysis of the level of intracellular MDA revealed that GPE pre-treatment of C2C12 cells reduced the production of MDA to a level similar to that in the positive control group (Figure 2(E)). These results suggest that the GPE attenuates the H_2_O_2_-mediated damage of cells.

### GPE enhance the activity of antioxidant enzyme in C2C12 cells

**Figure 3.**
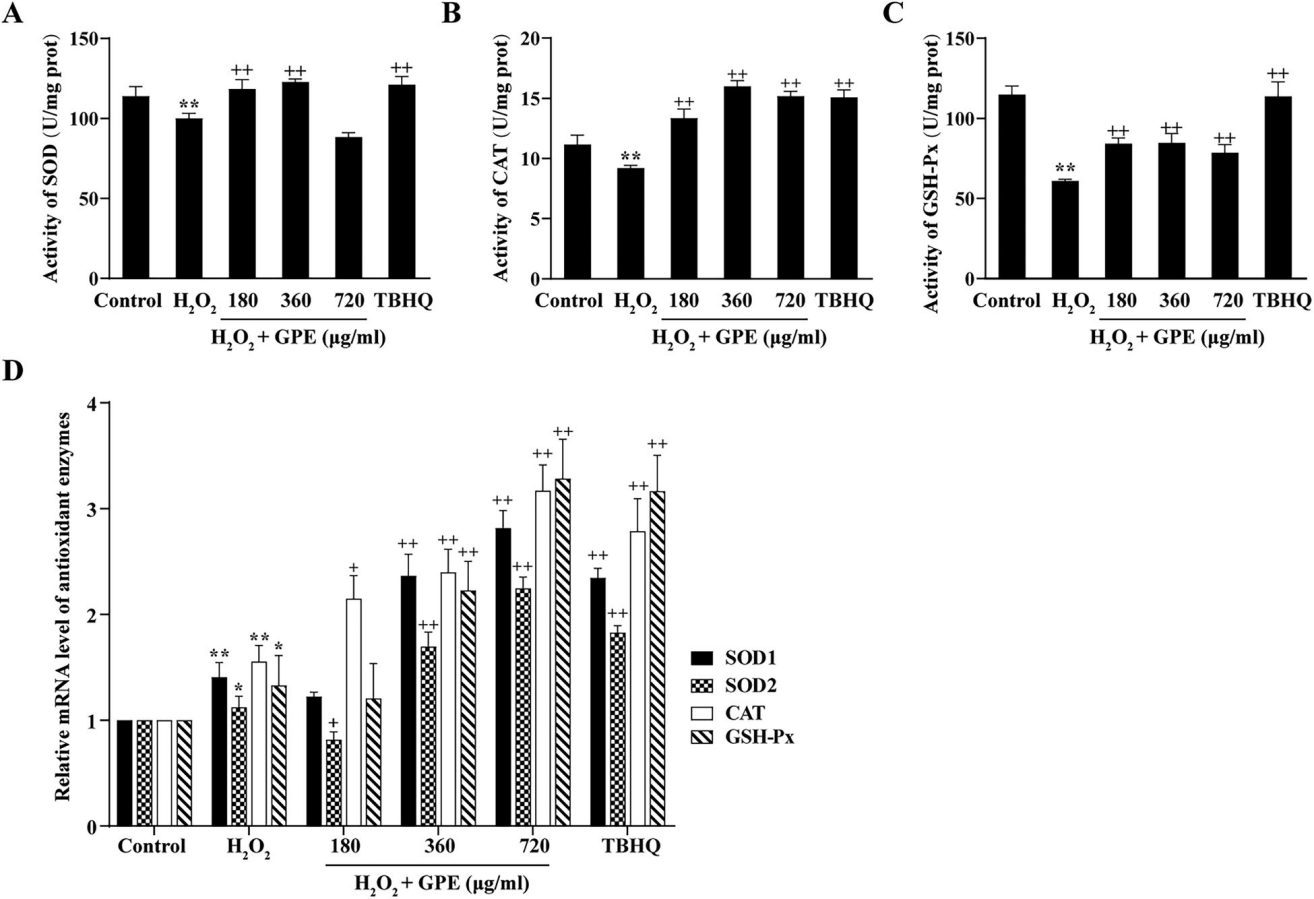
GPE enhances the activities of antioxidant enzymes in C2C12 cells. The activities of (A) SOD, (B) CAT, and (C) GSH-Px were analyzed by spectrophotometry. (D) RT-qPCR analysis of the mRNA expression level of the genes encoding the four antioxidant enzymes. *p < 0.05 and **p < 0.01 compared with control group, +*p* < 0.05 and ++*p* < 0.01 compared with H_2_O_2_-stimulated group.

To explore the molecular mechanism of the antioxidant effects of GPE, we measured the activity of the SOD, CAT, and GSH-Px antioxidant enzymes. There was an evident decline in the activities of these enzymes in C2C12 cells stimulated with H_2_O_2_, but the harmful effects were dramatically reversed for C2C12 cells pre-treated with GPE (Figure 3(A–C)). We next examined the mRNA expression levels of four antioxidant genes (*SOD1*, *SOD2*, *CAT* and *GSH-Px*) in four groups (Figure 3(D)). Expression of these genes was increased in cells pre-treated with GPE compared with that in cells not pre-treated prior to stimulation with H_2_O_2_. In addition, the expression levels of these genes in cells pre-treated with 360 or 720 μg/ml GPE dosage were comparable with those in the positive control group. These results indicated that GPE enhances the activity of antioxidant enzymes in C2C12 cells.

### GPE promotes NRF2 expression and nuclear translocation

**Figure 4.**
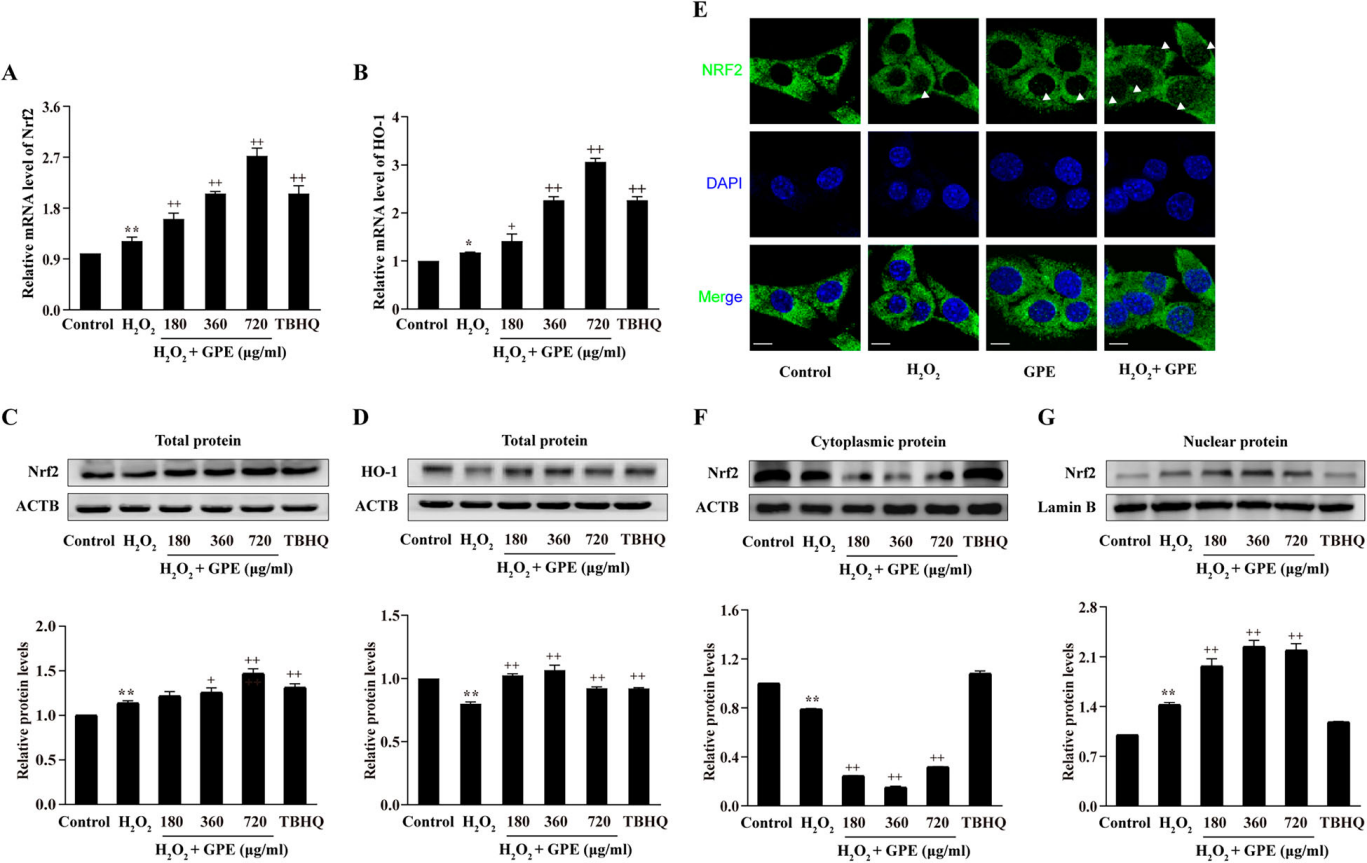
GPE promotes *NRF2* and *HO-1* expression and induces the nuclear-cytoplasmic shuttling of NRF2. Quantitative analysis of the mRNA expression levels of NRF2 (A) and HO-1 (B) relative to GAPDH by RT-qPCR. Western blot analysis of the total cellular protein expression levels of NRF2 (C) and HO-1 (D). (E) Immunofluorescence assay to determine NRF2 translocation. The white arrow heads indicate the nuclear localization of NRF2. Scale bar = 20 μm. Western blot analysis of the protein expression levels of NRF2 in the cytoplasmic (F) and nuclear (G) fractions. *p < 0.05 and **p < 0.01 compared with the control group, +*p* < 0.05 and ++*p* < 0.01 compared with the H_2_O_2_-stimulated group.

The NRF2-ARE antioxidant pathway regulates the expression of antioxidant proteins that protect against oxidative damage in cells. To explore the mechanisms of the antioxidant effects of GPE pre-treatment, we investigated the changes in the NRF2-ARE signaling pathway upon GPE and H_2_O_2_ treatment. As shown in Figure 4(A,B), RT-qPCR revealed that the mRNA levels of *NRF2* and *HO-1* in cells exposed to 360 and 720 μg/ml GPE were markedly higher than those in the H_2_O_2_ group. We also analyzed the protein expression levels of NRF2 and HO-1 by western blot and found that the same two concentrations of GPE used to pre-treat cells increased the expression of NRF2 and HO-1 proteins in C2C12 cells (Figure 4(C,D)), consistent with the mRNA expression levels. NRF2 nuclear translocation regulates the transcription of genes, which contain AREs in their promoters. Using immunofluorescence analysis, we found that GPE induced the nuclear-cytoplasmic shuttling of NRF2 under H_2_O_2_ stress (Figure 4(E)). We also investigated the shuttling of NRF2 between cell nucleus and cytoplasm by western blotting. As shown in Figure 4(F,G), NRF2 in the cytoplasm shuttled into the nucleus in the three GPE groups. Taken together, these results indicate that GPE pre-treatment of C2C12 cells improves the antioxidant ability of cells through the NRF2-ARE signaling pathway.

### Antioxidant effect of GPE is mediated by NRF2

**Figure 5.**
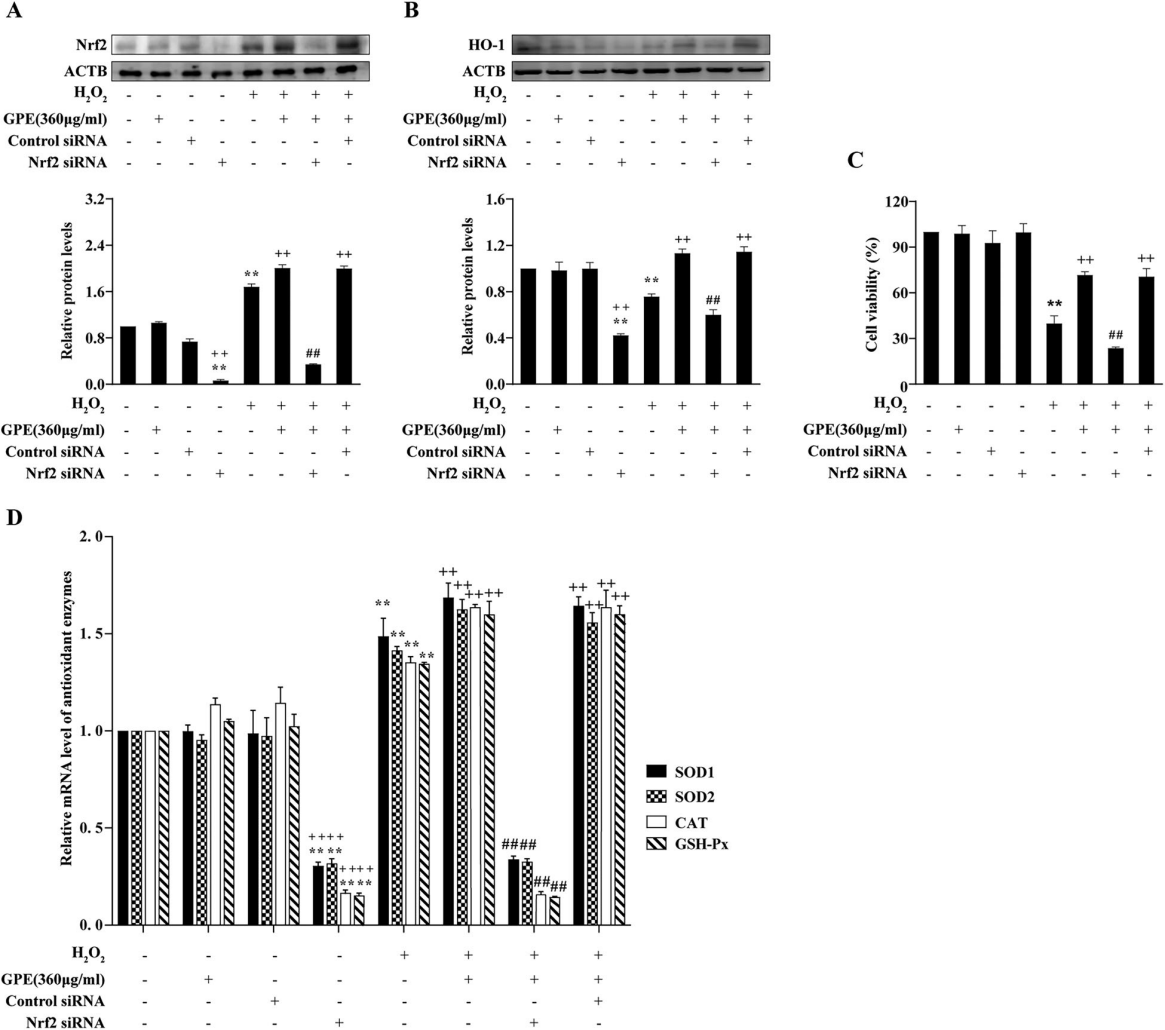
The antioxidant effect of GPE mediated by NRF2. (A and B) Western blot analysis of the protein expression levels of NRF2 and HO-1. (C) Cell viability assessed by the MTT reduction assay. (D) RT-qPCR analysis of the mRNA expression levels of the antioxidant enzymes following siRNA NRF2 knockdown. **p < 0.01 compared with control group, ++*p* < 0.01 compared with the H_2_O_2_-stimulated group, ##*p* < 0.01 compared with the H_2_O_2_ treated and GPE pre-treated groups.

To confirm the antioxidant effect of GPE via the NRF2-ARE signaling pathway, we established the *NRF2* gene knockdown model using siRNA transfection. As shown in Figure 5(A), NRF2 expression was sharply decreased in the cells transfected with NRF2 siRNA whether cells were pre-treated with GPE or not. HO-1 expression was also decreased by the transfection of NRF2 siRNA (Figure 5(B)). The MTT viability assay further demonstrated that GPE pre-treatment of cells enhances viability under H_2_O_2_ stimulation. This effect was blocked by NRF2 siRNA transfection (Figure 5(C)). The mRNA levels of the *SOD1*, *SOD2*, *CAT*, and *GSH-Px* antioxidant enzymes were remarkable up-regulated in cells pre-treated with GPE and then treated with H_2_O_2_ compared with those of cells not pre-treated (Figure 5(D)). These antioxidant genes were significantly down-regulated by the knockdown of *NRF2*. This evidence shows that the antioxidant effect of GPE is mediated by the activation of the NRF2-ARE signaling pathway.

### Schematic model of GPE protection against H_2_O_2_-induced cell damage through NRF2

**Figure 6.**
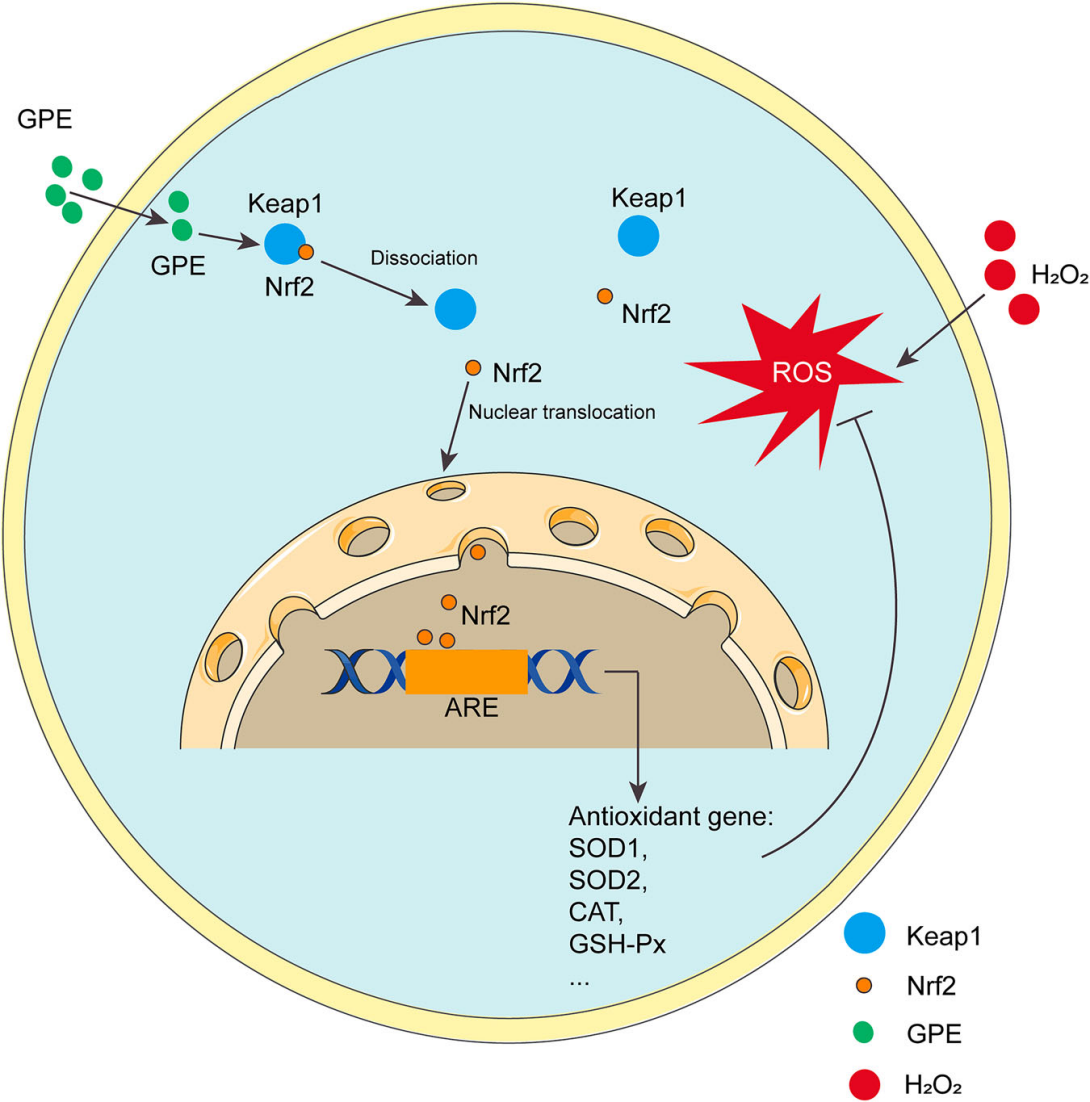
Schematic model of GPE protection against H_2_O_2_-induced cell damage through NRF2. GPE promotes NRF2 nuclear translocation to induce the expression of the antioxidant enzymes SOD1, SOD2, CAT, and GSH-Px. Their expression and activity eliminate the intracellular reactive oxygen species (ROS) and protect C2C12 cells against the induction of cell death by H_2_O_2_.

Using the C2C12 cell model, we demonstrated that GPE markedly promoted NRF2 nuclear translocation to induce the expression of antioxidant enzymes, which protected against H_2_O_2_-induced cell death (Figure 6).

## Discussion

Our bodies are continuously exposed to environmental stresses, such as toxic chemicals and oxidative stress, which elevate the intracellular levels of ROS. Excess ROS damages lipids, proteins, and DNA (Kregel and Zhang 2007; Uruno et al. 2016; Qi et al. 2017). Oxidative stress and other pathological conditions caused by ROS result in loss of myogenic ability, protein degradation, and increased cell death (Horie et al. 2015). Many studies have examined the medicinal benefits of antioxidants. However, little is known of traditional antioxidative preparations. The present findings demonstrate that GPE can protect C2C12 cells from oxidative stress caused by H_2_O_2_. GPE pre-treatment of C2C12 myoblasts significantly reduced the loss of cell viability due to H_2_O_2_. MDA, the final product of lipid peroxidation, is used as a biomarker to indicate the level of oxidative stress (Fu et al. 2013). In the present study, there was a decrease in MDA level in cells pre-treated with GPE. H_2_O_2_ increases DNA and cell membrane damage. This damage could be mitigated by the GPE treatment. The results demonstrate that GPE is an antioxidant that can protect cells from H_2_O_2_-induced damage.

SOD, CAT, and GSH-Px enzymes are important in protecting cells from oxidative stress due to free radicals (Kim et al. 2015). An increasing number of studies have described polypeptides related to antioxidant enzymes and their ability to protect cells against oxidative stress. For example, corn gluten meal-derived peptides can enhance the effects of SOD, CAT, and GSH-Px activity in HepG2 cells under oxidative stress and increase cell viability (Wang et al. 2016). Brevinin-2R peptide reportedly increased SOD and GSH-Px activities and enhanced A549 cell viability during oxidative stress (Ghodsi-Moghadam and Asoodeh 2019). In the present study, we analyzed the activity of the SOD, CAT, and GSH-Px antioxidant enzymes to examine their beneficial effects in the resistance of GPE pre-treated cells to oxidative stress. H_2_O_2_ reduced the activity of the antioxidant enzymes when the cells were not pre-treated with GPE. In contrast, GPE pre-treatment enhanced the activities of the enzymes at the mRNA and protein levels.

The NRF2 signaling pathway is pivotal in antioxidant activity. KEAP1 rapidly ubiquitinates and degrades NRF2 in the cytoplasm. The resulting suppressed transcriptional activity of NRF2 induces the activity of a set of genes encoding antioxidant and detoxification enzymes. When cells are exposed to oxidative or electrophilic stress, the cysteine residues of Keap1 are modified, which destroys the ability to ubiquitinate NRF2 (Murphy et al. 2018). The translocation of NRF2 from the cytoplasm to cell nucleus is a core step to activate the expression of the downstream antioxidant genes (Bellezza et al. 2018). *HO-1* gene expression is mainly regulated by the NRF2-ARE pathway. Increasing evidence supports the protective effect of the enzyme against oxidative stress-induced cell death and various tissue injury (Jeong et al. 2019). Extracts from natural medicines have antioxidative and cellular protective effects involving the activation of the NRF2-ARE signaling pathway and increased expression of (Zhang et al. 2019). To detect the anti-oxidation mechanism of GPE, we tested the protein and mRNA expression levels of NRF2 and HO-1. GPE increased the protein and mRNA expressions of NRF2 and promoted its cytoplasmic and nuclear translocation, which induced the expression of HO-1. The advantageous effects of GPE, including increasing the expression of antioxidant enzymes and cell viability, disappeared when NRF2 was silenced in C2C12 cells. These findings imply that GPE might protect cells from damage by increasing the expression of antioxidant enzymes induced by the activation of the NRF2-ARE signal pathway. In addition, NRF2 gene knockdown in C2C12 cells clearly demonstrated that the antioxidant effects of GPE were mediated by the NRF2-ARE pathway.

In summary, GPE markedly induces NRF2-mediated antioxidant enzyme expression, which contributes to the cellular defense mechanism against oxidative stress. Although the definite molecular mechanisms require further investigation, the present findings indicate the potential therapeutic value of GPE as an antioxidant agent.
